# Incidental Radiographic Discovery of a Screw in a Primary Molar: An Unusual Case Report in a 6 Year Old Child

**DOI:** 10.1155/2013/296425

**Published:** 2013-06-26

**Authors:** Farhin Katge, Sajjad Mithiborwala, Thejokrishna Pammi

**Affiliations:** Department of Pedodontics & Preventive Dentistry, Terna Dental College, Sector 22, Plot No. 12, Nerul (W), Navi Mumbai 400706, Maharashtra, India

## Abstract

Dentists often find foreign bodies in the primary dentition of children who habitually place objects in their mouths. The objects are frequently embedded in exposures that result from carious or traumatic lesions or from endodontic procedures that have been left open for drainage. Such bodies are often detected on routine radiographs and, less frequently, during clinical examination. We report a case of a 6-year-old boy who had inadvertently embedded a screw in his mandibular right first primary molar and had forgotten about it until it became symptomatic. The screw was impacted in the exposed pulp chamber due to a large carious lesion in the affected molar. This case report considers the possible medical and dental consequences of placing foreign bodies in the mouth.

## 1. Introduction

Many children are in the habit of exploring various objects in the oral cavity that can cause hard or soft tissue injuries. This practice may result in inadvertent insertion of foreign bodies within the pulp chamber or root canal [1]. Foreign objects are often discovered in the primary dentition during radiological examination. Radiographic examination assists in the determination of the number, type, composition, and position of the foreign object(s). Till date only two such cases of a screw impacted in a primary molar have been reported [2, 3]. 

## 2. Case Report

**Figure 1:**
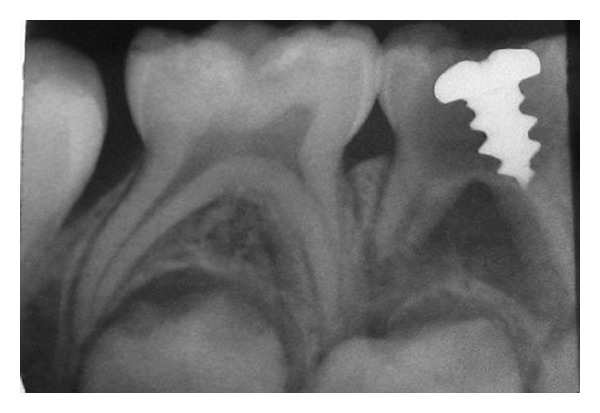
IOPA showing metallic screw in the pulp chamber of #84 with intact pulpal floor.

**Figure 2:**
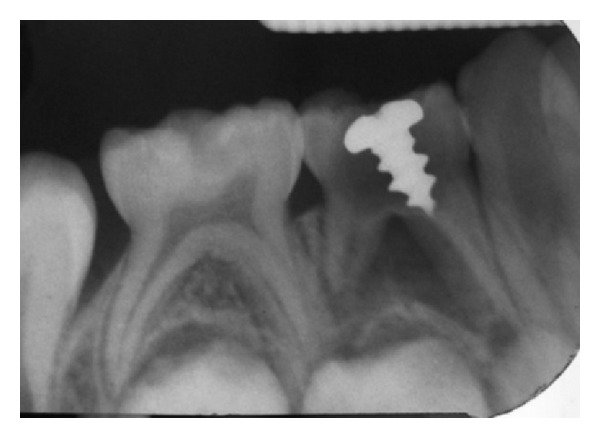
IOPA showing intact furcal area with no evidence of interradicular bone loss.

 A 6 year old boy reported to the Department of Pedodontics, Terna Dental College, Navi Mumbai, India, complaining of pain in lower right back region of jaw, since 3–5 days. Intraoral clinical examination revealed deep occlusal caries and intraoral draining sinus in relation to #84. Vestibular tenderness and mobility were absent. An intraoral periapical radiograph of the tooth revealed presence of a linear radiopaque object (metallic screw) 6.5 × 4 mm in dimension, which was embedded in the pulp chamber of #84 (Figures 1 and 2). A clinical history revealed that the patient habitually placed metal objects in his mouth; on one occasion, the screw had become lodged in the cavitated tooth. Several attempts by the child to retrieve it had proven futile. The child had not reported this incident to his parents for fear of punishment and had soon forgotten about it. A diagnosis of chronic dentoalveolar abscess with 84 was made. The treatment plan involved removal of foreign body from the pulp chamber of #84 followed by pulpectomy and stainless steel crown cementation.

**Figure 3:**
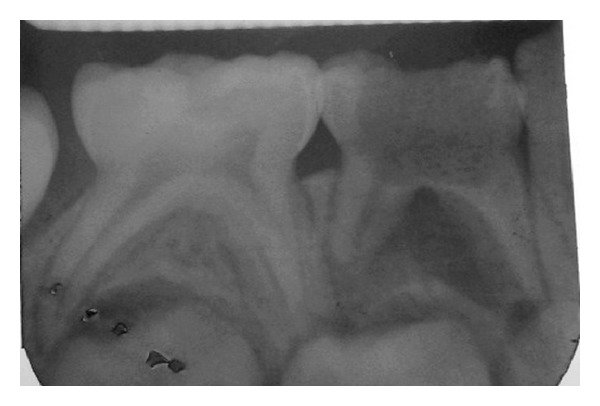
IOPA after retrieval of the metallic screw.

 Clinical procedure was embarked with hand excavation of the large carious lesion. Following excavation of the carious lesion the site was flushed with isotonic saline which then revealed the metallic screw. With the help of piezoelectric ultrasonics (Dentsply Tulsa Dental Specialties, USA) [4] at low intensity the screw was very slowly disengaged from the pulpal chamber taking care that the thin buccal and lingual walls did not fracture (Figure 3). 

**Figure 4:**
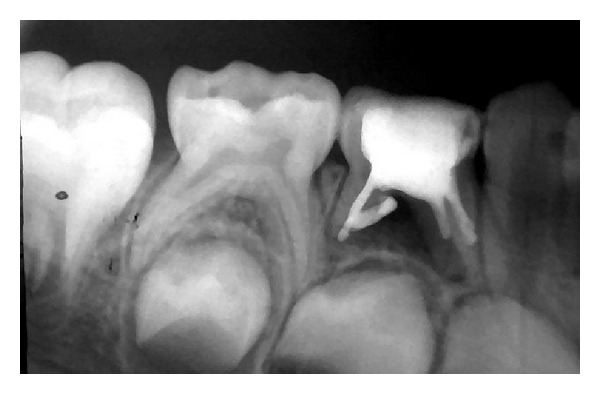
Obturation of the #84.

After the retrieval of the screw, the tooth was clinically evaluated for the signs of perforation of the pulpal floor. Careful biomechanical preparation of the root canals was done to avoid lateral root perforation. Concomitant copious irrigation with endodontic irrigants was done to debride the root canals. Following obturation with Vitapex (Neo Dental International, Inc., USA) the access cavity was restored with Ketac Molar (3M ESPE, USA) (Figure 4). The crown was then cemented with stainless steel crown (3M ESPE, USA) with luting cement.

The patient was kept under systemic antibiotic therapy, which was started one day prior to the clinical procedure (Amoxicillin 250 mg; Metronidazole 200 mg; and Tab Ibugesic kid 20 mg TID for 5 days). A tetanus toxoid vaccine (tetanus vaccine (adsorbed) Ip., Biological E. Limited, Hyderabad, India) was administered after the obturation of the tooth. One month recall examination showed complete healing of the intraoral sinus.

## 3. Discussion

Self-oral exploration, play while eating, imitation of peers or older siblings performing a similar behaviour, and attempts to relieve chronic irritation coupled with fear of dentistry are factors that may prompt children to place foreign objects in the mouth. A review of the literature reveals numerous reports describing the various foreign objects that have been inserted in the exposed pulp chambers or root canals. Most such case reports have dealt with anterior teeth. Objects such as wooden tooth picks, straws, pins, needles, a pencil tip, plastic objects, toothbrush bristles, crayons, beads, paper clip, and stapler pins [5–9] have been placed into the root canals of maxillary anteriors in an attempt to remove food plugs [10–12].

Within the oral cavity, foreign bodies may be embedded in the soft or hard tissue. Objects impacted within the periodontium are a potential source of infection and may lead to edema, hemorrhage, and abscess formation. In addition, foreign bodies in primary teeth can lead to the perforation of the pulp chamber floor space and possible trauma to the developing permanent dentition, depending on developmental stage. Trauma to the tooth in the initial stages of odontogenesis can destroy the permanent tooth bud completely or may result in disorganization of the tooth germ, forming a complex odontoma.

A force of lesser magnitude may result in a geminated and/or a hypoplastic successor tooth. Furthermore, the presence of a foreign body may impede the eruption of the underlying permanent tooth, resulting in ectopic or failed eruption. Such failure of the underlying tooth to erupt due to an overlying mechanical obstruction at the time of root formation may alter the angulation of its root, leading to dilaceration. A foreign object lodged in the tooth for a lengthy period of time is a potential source of infection and can lead to cyst formation. Therefore, the eruption of the permanent tooth should be closely and regularly monitored so that any anomaly can be detected at an incipient stage and treatment rendered accordingly.

The management of a foreign body impaction in a tooth depends on the location, accessibility, stage of tooth formation, restorability of the tooth, the patient's age, and level of cooperation. However, very few such cases have been reported in posterior teeth. Prabhakar et al. reported embedment of a screw in a permanent mandibular first molar; while Nadkarni et al. described retrieval of a needle fragment from the palatal root canal of a permanent maxillary first molar [13, 14]. A meticulous review of the literature reveals that our case report is unique as the tooth in question is a primary lower right first molar, and no similar report till date has been published.

A conventional practice employed during emergency root canal treatment involves leaving the pulp chamber open where pus continues to discharge through the canal and cannot be dried within a reasonable period of time. Weine recommends that the patient remains in office with a draining tooth for an hour or even more and finally ending the appointment by sealing the access cavity. With the access cavity closed, no new strains of microorganisms are introduced, and food debris and foreign body lodgement within the tooth can be avoided [15].

A radiograph can be of diagnostic significance especially if the foreign body is radiopaque. Mcauliffe et al. summarized various radiographic methods to be followed to localize a radiopaque foreign object as parallax views, vertex occlusal view, triangulation techniques, and stereo radiography and tomography. Parallax technique involves 2 radiographs taken at different horizontal angles with the same vertical direction. Due to parallax the objects appear to travel in the same direction as tube shifts, and the object closer to tube appears to move in opposite direction (the so-called Same Lingual Opposite Buccal—SLOB). Vertex occlusal view is no longer favored because of relatively high radiation exposure to the lens of the eye and because the primary beam is aimed towards the abdomen. Triangulation is by the use of two views right angle to one another. Interpretation is difficult because of the superimposition of the other incisor teeth over the root. Stereographic views and tomography were not considered due to minimal availability of these facilities in dental operatory. Specialized radiographic techniques such as radiovisiography and 3D CAT scans can play a role in localization of these foreign objects inside the root canal [16].

## 4. Conclusion

 This case report would like to highlight the need of dental surgeons to establish good rapport with children, so that the history of foreign body impaction is not overlooked. Also evident is the need for an early radiological examination in cases of suspected “mouthing” of a foreign body. Moreover, teeth with traumatically or cariously exposed pulp should be coronally sealed whenever possible to avoid such complications. Moreover, a dire need exists to counsel parents in adopting basic home safety measures for children and vigilantly avoiding their having access to foreign objects within easy reach. In the present case, conservative method has been employed and extraction of the tooth prevented, since the patient had reported timely.
